# Correction: Solvent-controlled synthesis of multicolor photoluminescent carbon dots for bioimaging

**DOI:** 10.1039/c9ra90060h

**Published:** 2019-08-23

**Authors:** Yang Yan, Longyu Xia, Lan Ma

**Affiliations:** Department of Chemistry, Tsinghua University Beijing 100084 P. R. China; Division of Life Science and Health, Tsinghua University Graduate School at Shenzhen Shenzhen 518055 P. R. China malan@sz.tsinghua.edu.cn +86-0755-26033033

## Abstract

Correction for ‘Solvent-controlled synthesis of multicolor photoluminescent carbon dots for bioimaging’ by Yang Yan *et al.*, *RSC Adv.*, 2019, **9**, 24057–24065.

The authors regret that the contributions of the authors were not correctly indicated in the original article. Yang Yan should be designated as the sole first author, and therefore the footnote stating “The two authors contributed equally” was in error. The correct author list is as shown above.

The Royal Society of Chemistry apologises for these errors and any consequent inconvenience to authors and readers.

## Supplementary Material

